# Supplementary material for: Educational interventions delivered to prescribing advisers to influence primary care prescribing: a very low-cost pragmatic randomised trial using routine data from OpenPrescribing.net
Source: BMC Health Serv Res. 2025 Feb 25;25:308. doi: 10.1186/s12913-024-11575-y (PMC11854403; doi:10.1186/s12913-024-11575-y)
Supplement: Supplementary file 1 — Supplementary Material 1. [file 12913_2024_11575_MOESM1_ESM.pdf]

Low Priority Prescribing guidance

Brian Mac Kenna/Bhavana Reddy – NHS England

Autumn 2018

Declarations

NHS England: Specialist Pharmacist Adviser & Medicines Data Clinical Lead – Medicines & Diagnostics Policy Unit

University of Oxford, EBM Datalab - Honorary Research Fellow Pharmacist

Other declarations: http://www.whopaysthisdoctor.org/doctor/491

Declarations

NHS England: Specialist Pharmacist Adviser – Medicines and Diagnostics Policy Unit

Newcastle Trust: Head of Prescribing Support, Regional Drug and Therapeutics Centre.

No other declarations: http://www.whopaysthisdoctor.org/doctor/513

There is growing pressure on the NHS drugs bill

NHS England

Due to people living longer, more complex and innovative medicines being developed, and more specialist medicines being used

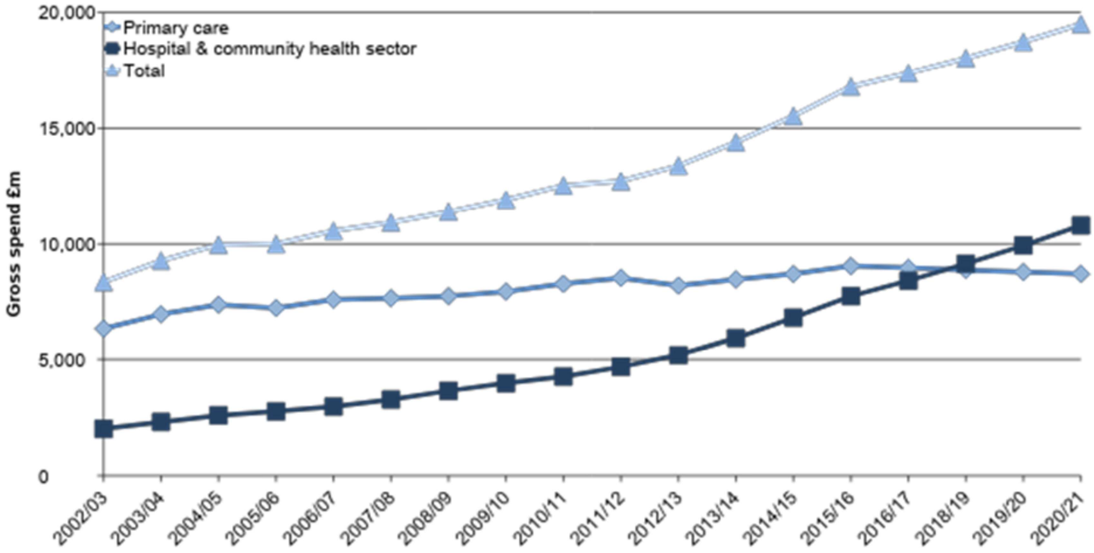

Medicines costs at list price (excl. VAT) before any discounts

www.england.nhs.uk

- Overall medicines spend 2016/17 was £17.4bn, an increase of 33.7% from £13bn in 2010/11
- Cost of medicines prescribed and dispensed in primary care rose from £8.6bn in 2010/11 to £9.0bn in 2016/17, a rise of 3.6%
- Cost of medicines used in hospitals increased from £4.2bn in 2010/11 to £8.3bn in 2016/17, a rise of 98.3%

Medicines are an important part of NHS care and help many people to get well

NHS England

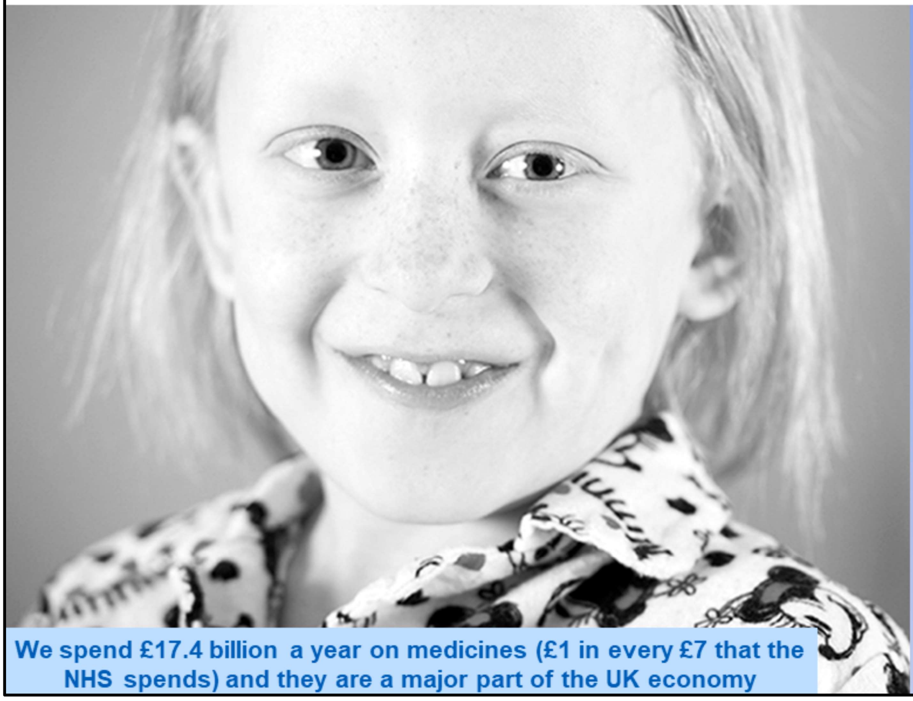

We spend £17.4 billion a year on medicines (£1 in every £7 that the NHS spends) and they are a major part of the UK economy

However, quality, safety and increasing costs continue to be issues…

- Around 5-8% of hospital admissions are medicines related, many preventable
- Bacteria are becoming resistant to antibiotics through overuse which is a global issue
- Up to 50% of patients don't take their medicines as intended, meaning their health is affected
- Use of multiple medicines is increasing – over 1 million people now take 8 or more medicines a day, many of whom are older people

How to get maximum value from the spend on medicines

NHS England

Value is… measurable improvement in patient outcomes while maintaining an affordable medicines bill

Making sure **patients get access to and choice of** the most effective treatments, and the outcomes that matter to them

Improving the quality (safety, clinical effectiveness, patient experience) **of prescribing and medicines use**

Making how we **purchase and supply medicines more efficient**, while ensuring the NHS retains its position as a world-leader in medicines

www.england.nhs.uk

The Medicines Value Programme has been set up to respond to these challenges

NHS England

Following the Next Steps on the NHS Five Year Forward View and Carter Report

The NHS wants to help people to get the best results from their medicines – while achieving best value for the taxpayer

Savings will be reinvested in improving patient care and providing new treatments to grow the NHS for the future

1. **The NHS policy framework that governs access to and pricing of medicines**
2. **The commercial arrangements that influence price**
3. **Optimising the use of medicines**
4. **Developing the infrastructure to support an efficient supply chain**

A whole system approach….

- NHS England, NHS Improvement, NHS Digital, Health Education England
- Regional offices link with STPs, ICSs, CCGs, and providers
- Nationally coordinated with AHSNs, Getting It Right First Time, NHS Right Care and NHSCC

Low Priority Prescribing guidance: Why?

NHS England

NHS Clinical Commissioners

The independent collective voice of clinical commissioning groups

- Last year 1.11 billion prescription items were dispensed in primary care at a cost of £9.17billion.
- It is important that the NHS achieves the greatest value from the money that it spends. We know that across England there is significant variation on what is being prescribed, at what price and to whom.
- In addition, patients often receive medicines which have been proven to be ineffective, or for which there are other more effective and/or cheaper alternatives.
- This object of this work is to support Clinical Commissioning Groups (CCGs) to remove unwarranted variation where it exists, and to provide consistent national clinical commissioning advice to inform local decisions which need to be taken to support effective prescribing practices

www.nhscc.org

A networked organisation of NHS CONFEDERATION

Data is taken from the NHS Digital Prescribing Cost Analysis data https://digital.nhs.uk/data-and-information/publications/statistical/prescription-cost-analysis/prescription-cost-analysis-england-2017

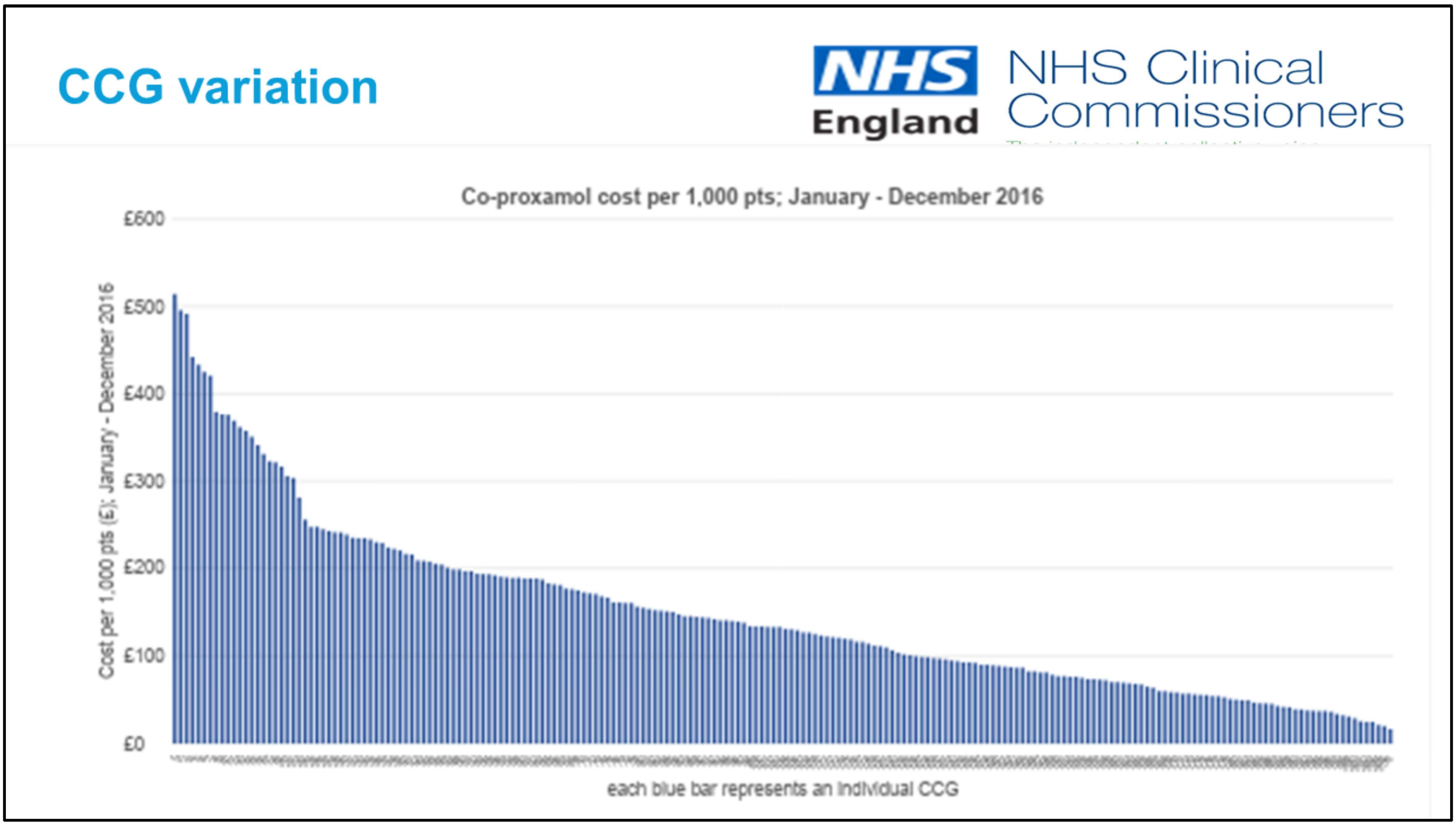

Data is taken from the NHS Digital Prescribing Cost Analysis data https://digital.nhs.uk/catalogue/PUB23631 Data for individual CCGs and GP practices is available from PrescQIPP CIC (see final slide)

This time period is selected to demonstrate the variation when the guidance was released.

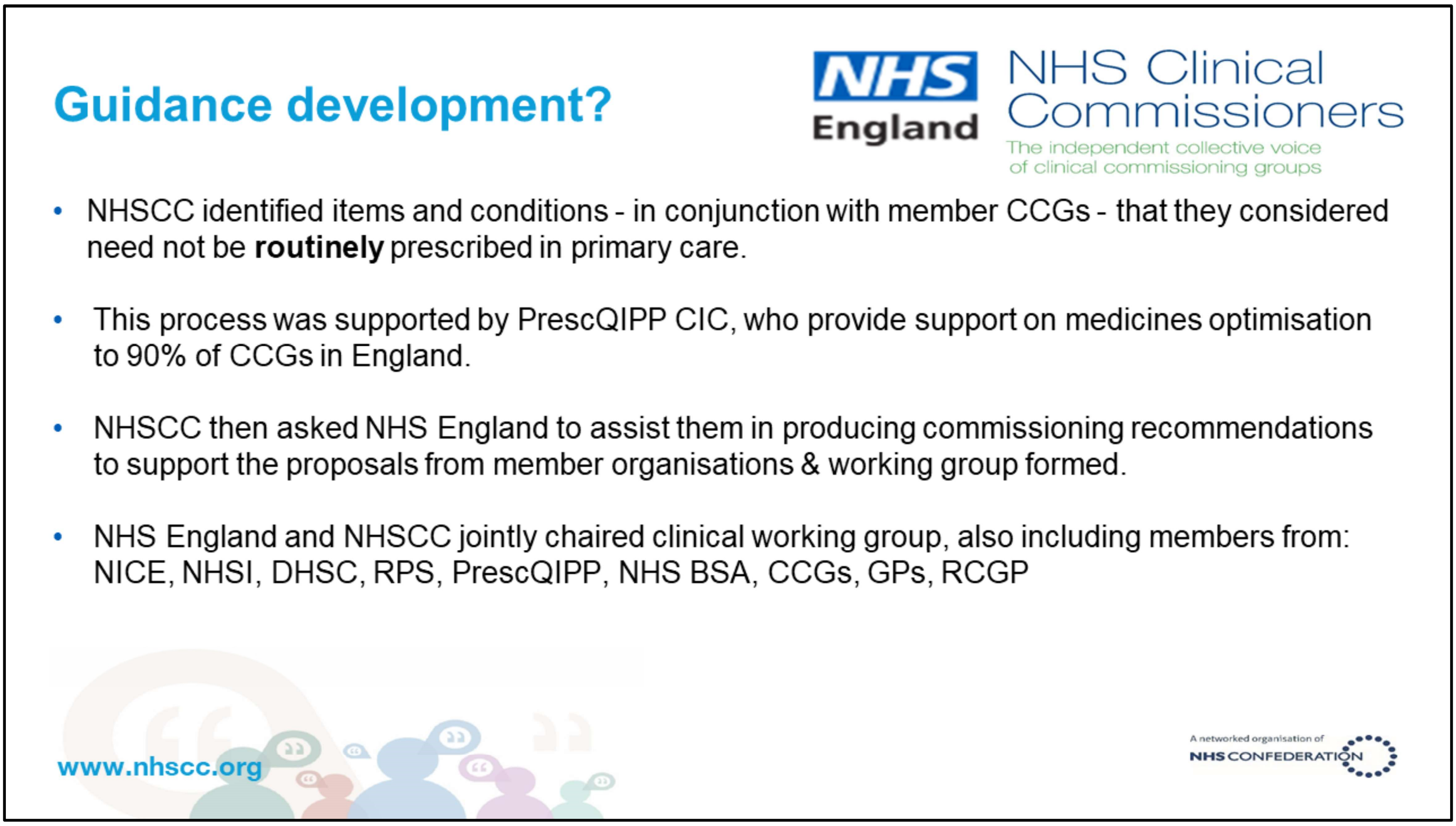

NHS Clinical Commissioners are the independent membership organisation for CCGs, providing their collective voice, facilitating shared learning and delivering networking opportunities for CCG members.
NHS England leads the National Health Service (NHS) in England. We set the priorities and direction of the NHS and encourage and inform the national debate to improve health and care.
PrescQIPP CIC (Community Interest Company): PrescQIPP are an NHS funded not for-profit organisation that supports quality, optimised prescribing for patients. They produce evidence-based resources and tools for primary care commissioners, and provide a platform to share innovation across the NHS.

A consultation report can be viewed at https://www.england.nhs.uk/medicines/items-which-should-not-be-routinely-prescribed/

Low Priority Prescribing guidance

NHS England

NHS Clinical Commissioners
The independent collective voice of clinical commissioning groups

- Both sets of draft proposals were subject to a 3 month public consultation which resulted in 5,516 online responses (18 items) & 2,638 (minor conditions) + face to face, focus group and written correspondence.

- CCG Commissioning guidance was approved by the NHS England board and issued to CCGs to have regard to in development of local prescribing policies.

www.nhscc.org

A networked organisation of NHS CONFEDERATION

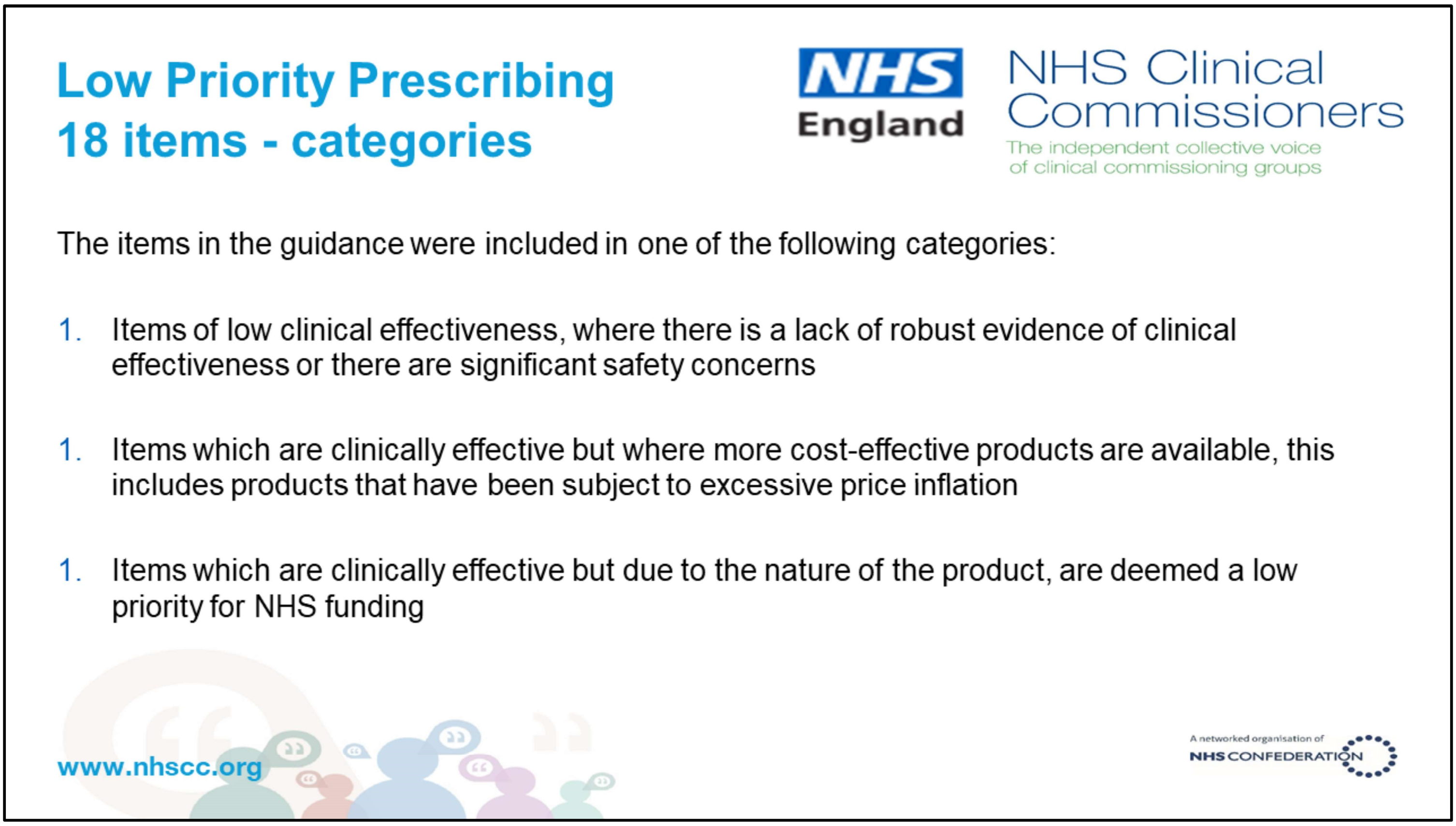

CCGs would be expected to take this into account in formulating local polices, and for prescribers to reflect local policies in their prescribing practice.

Recommendations

NHS England

NHS Clinical Commissioners
The independent collective voice of clinical commissioning groups

- Advise CCGs that prescribers in primary care should not initiate {item} for any new patient.
- Advise CCGs to support prescribers in de-prescribing {item} in all patients and where appropriate, ensure the availability of relevant services to facilitate this change.
- Advise CCGs that if, in exceptional circumstances, there is a clinical need for the item to be prescribed in primary care, this should be undertaken in a cooperation arrangement with a multi-disciplinary team and/or other healthcare professional.
- Advise CCGs that all prescribing should be carried out by a specialist.
- Advise CCGs that this item should not be routinely prescribed in primary care but may be prescribed in circumstances such as {item}.

www.nhscc.org

A networked organisation of NHS CONFEDERATION

Each item included was assigned at least one of the above recommendations.

In this context, "exceptional circumstances" should be interpreted as: Where the prescribing clinician considers no other medicine or intervention is clinically appropriate and available for the individual.

Low Priority Prescribing
18 items

NHS England NHS Clinical Commissioners
The independent collective voice of clinical commissioning groups

Low evidence or safety	Cost-effectiveness	Low priority for funding
• Co-proxamol<br>• Dosulepin<br>• Glucosamine and Chondroitin<br>• Herbal Treatments<br>• Homeopathy<br>• Lidocaine Plasters<br>• Lutein and Antioxidants<br>• Omega-3 Fatty Acid Compounds<br>• Rubefacients (excluding topical NSAIDs)	• Prolonged-release Doxazosin<br>• Immediate Release Fentanyl<br>• Liothyronine<br>• Oxycodone and Naloxone Combination Product<br>• Paracetamol and Tramadol Combination Product<br>• Perindopril Arginine<br>• Tadalafil (Once daily)<br>• Trimipramine	• Travel vaccines

www.nhscc.org

A networked organisation of NHS CONFEDERATION

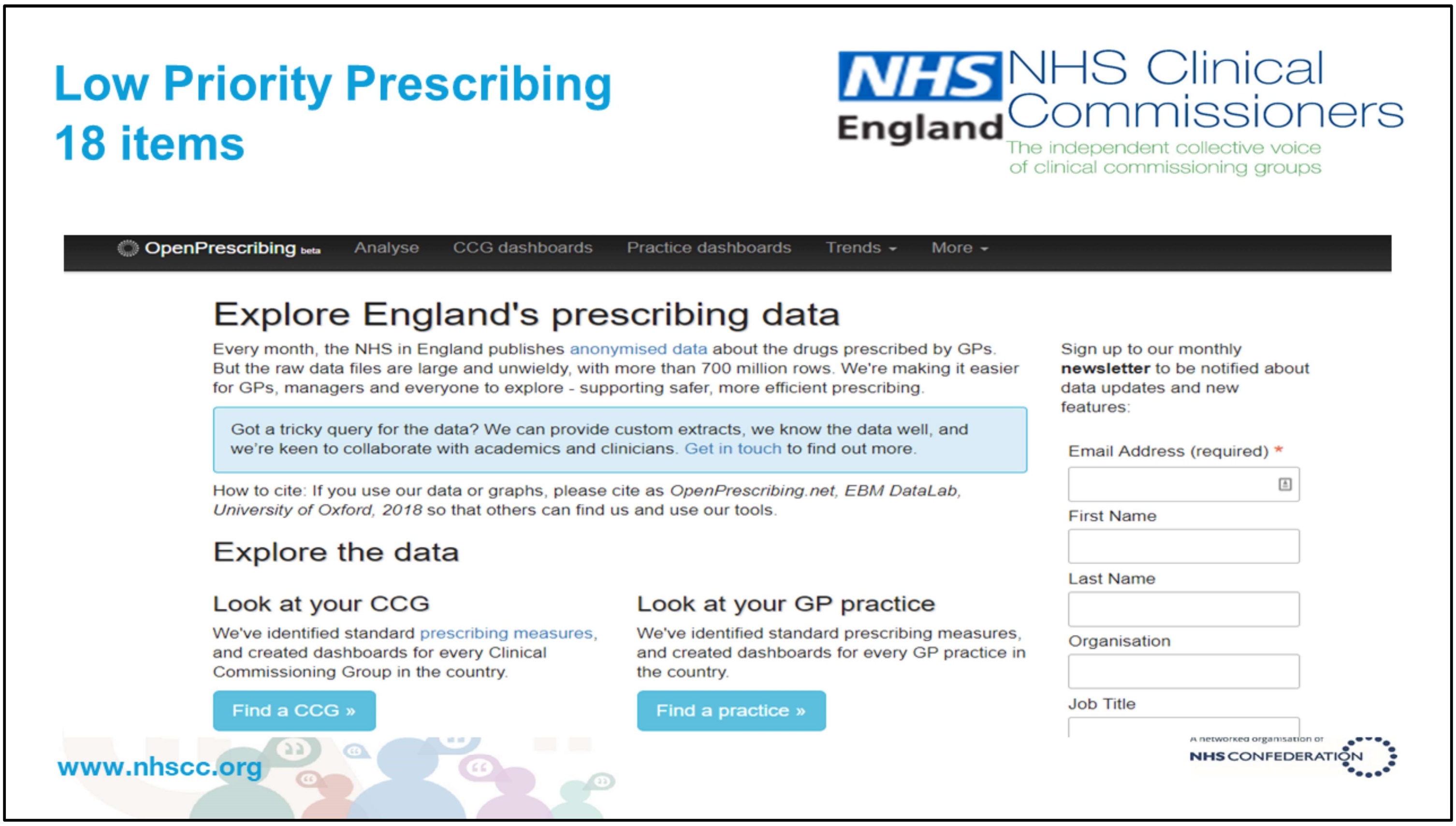

Demonstrate OpenPrescribing. Analyse, tariff viewer, GP dashboards, CCG dashboards, alert system.

FAQ: Why aren’t we using ePACT2? ePACT2 contains very detailed data for use by meds opt teams. It is not targeted at GPs and front line clinicians. Secondly getting passwords, unsure what facilities will be available in each venue means it is less suitable for our purposes.
Also mention PrescQIPP data availability.

Implementation

ePACT2 Dashboard

NHS England | NHS Clinical Commissioners

Secure | https://bi2-a437212.analytics.em3.oraclecloud.com/analytics/saw.dll?Portal&PortalPath=%2Fcompan

Apps | ESR Programme Sup

ORACLE Business Intelligence Cloud Service

Restricted Items

Your feedback is valued and will help us to improve our system;
Please email us at nhsbsa.informationsystems@nhs.net

Please select dashboard page:- Overview | CCG Summary | Practice Summary | Organisation Summary | Datasets | Supplementary Information

Criteria Selection

* Select time period: 201712;201801;2018

Apply | Reset

Dashboard Overview

Overview

This dashboard focuses on a list of 18 treatments, drawn up with family doctors and pharmacists, deemed to be ineffective, over-priced and of low clinical value.

Work focused on developing guidelines for an initial list of 18 products which fell into one or more of the following categories:
Products of low clinical effectiveness, where there is a lack of robust evidence of clinical effectiveness or there are significant safety concerns;
Products which are clinically effective but where more cost effective products are available, including some products that have been subject to excessive price inflation;
Products which are clinically effective but due to the nature of the product are deemed a low priority for NHS funding.

In the majority of cases there are other more effective, safer and/or cheaper alternatives available to the treatments that NHS England is recommending should not be routinely prescribed in primary care.

Interpretation of the reports/data

The reports show the prescribing of products split by the 18 medicine categories, with data collated over the selected time period.

The data for each medicine category should be interpreted alongside the recommendations for each item as set out in the guidance document (see link under "Supplementary Information")

Please note that anytime patient figures are reported, a single patient may appear in the counts for multiple different medicines.

Future developments

This dashboard will be subject to further development activities in the future to incorporate additional data and functionality identified both internally and in response to user feedback.

Initial development plans include the addition of cost/item growth to allow the tracking of changes from month to month.

Summary Data

Select dataset to view: National Data

National Data (Dec 17 - Feb 18)

Medicine Category	Items	Net Ingredient Cost (£)	Actual Cost (£)	No. of Identifiable patients
Co-Proxamol	6,605	1,211,914.49	1,124,323.85	2134
Dosulepin	187,600	394,997.81	372,060.78	60337
Glucosamine and Chondroitin	1,675	29,789.19	27,794.07	637
Herbal Medicines	1,637	22,706.11	21,124.94	820
Homeopathy	920	15,901.21	15,231.42	565
Immediate Release Fentanyl	7,705	2,347,691.70	2,177,073.88	1425
Lidocaine Plasters	53,999	4,130,362.45	3,831,419.48	27018
Liothyronine	15,651	6,777,140.92	6,284,800.51	6657
Lutein and Antioxidants	6,184	50,802.83	47,557.25	2387
Omega-3 Fatty Acid Compounds	38,884	1,208,133.58	1,121,583.51	15209
Once Daily Tadalafil	45,386	2,907,471.88	2,696,926.22	20430
Oxycodone and Naloxone Combination Product	14,568	1,038,648.90	963,456.76	4128
Paracetamol and Tramadol Combination Product	20,986	170,861.31	159,391.01	9000
Perindopril Arginine	26,331	331,220.77	307,350.56	11299
Prolonged-release Doxazosin	136,468	1,567,050.15	1,456,691.77	52899
Rubefacients (excluding topical NSAIDs)	222,356	1,240,053.85	1,152,456.60	128259
Travel Vaccines	29,040	499,192.04	462,906.19	4723
Trimipramine	11,051	4,075,405.81	3,778,914.07	3438
Grand Total	827,049	28,019,345.00	26,001,062.87	345951

FAQ: Why aren't we using ePACT2? ePACT2 contains very detailed data for use by meds opt teams. It is not targeted at GPs and front line clinicians. Secondly getting passwords, unsure what facilities will be available in each venue means it is less suitable for our purposes.

There is a detailed dashbord here with details of patient numbers etc.

Also mention PrescQIPP data availability.

Items for Pain

NHS England

NHS Clinical Commissioners
The independent collective voice of clinical commissioning groups

- Co-proxamol
- Fentanyl (immediate release)
- Glucosamine and Chondroitin
- Lidocaine Plasters
- Oxycodone and Naloxone Combination product
- Paracetamol and Tramadol Combination product
- Rubefacients

www.nhscc.org

A networked organisation of NHS CONFEDERATION

Co-proxamol

NHS England

NHS Clinical Commissioners

The independent collective voice of clinical commissioning groups

- Co-proxamol was a pain-killer which was previously licensed in the UK until being fully withdrawn from the market in 2007 due to safety concerns.
- All use in the UK is now on an unlicensed basis.
- Since the withdrawal, further safety concerns have been raised which have resulted in co-proxamol being withdrawn in other countries.
- Since the withdrawal, 135 deaths related to poisoning have occurred where co-proxamol was mentioned on the death certificate.
- Co-proxamol is categorised as an item of low clinical effectiveness, where there is a lack of robust evidence of clinical effectiveness or there are significant safety concerns.

www.nhscc.org

A networked organisation of NHS CONFEDERATION

Supporting References and Resources

https://www.gov.uk/drug-safety-update/co-proxamol-withdrawal-reminder-to-prescribers

https://www.gov.uk/drug-safety-update/-dextro-propoxyphene-new-studies-confirm-cardiac-risks

https://www.prescqipp.info/resources/category/90-co-proxamol

https://www.ons.gov.uk/peoplepopulationandcommunity/birthsdeathsandmarriages/deaths/datasets/deathsrelatedtodrugpoisoningenglandandwalesreferencetable

Co-proxamol Recommendations

NHS England
NHS Clinical Commissioners
The independent collective voice of clinical commissioning groups

- Advise CCGs that prescribers in primary care should not initiate co-proxamol for any new patient.
- Advise CCGs to support prescribers in de-prescribing co-proxamol in all patients and, where appropriate, ensure the availability of relevant services to facilitate this change.

No routine exceptions have been identified.

www.nhscc.org

A networked organisation of NHS CONFEDERATION

At this point display the dashboard data from OpenPrescribing for the CCG in question.
Explore the data
Discuss strategies to further reduce prescribing

Other items

Mental Health

- Dosulepin
- Trimipramine

Please note that these medicines may be used off-label for indications not covered in this presentation.

Dosulepin

NHS England

NHS Clinical Commissioners

The independent collective voice of clinical commissioning groups

- Dosulepin, formerly known as dothiepin, is a tricyclic antidepressant.
- NICE CG90: Depression in Adults has a "do not do" recommendation: *"Do not switch to, or start, dosulepin because evidence supporting its tolerability relative to other antidepressants is outweighed by the increased cardiac risk and toxicity in overdose."*
- Dosulepin is categorised as items of low clinical effectiveness, where there is a lack of robust evidence of clinical effectiveness or there are significant safety concerns.

www.nhscc.org

A networked organisation of NHS CONFEDERATION

Supporting References and Resources

https://www.nice.org.uk/Guidance/CG90

https://www.prescqipp.info/resources/category/313-dosulepin-drop-list

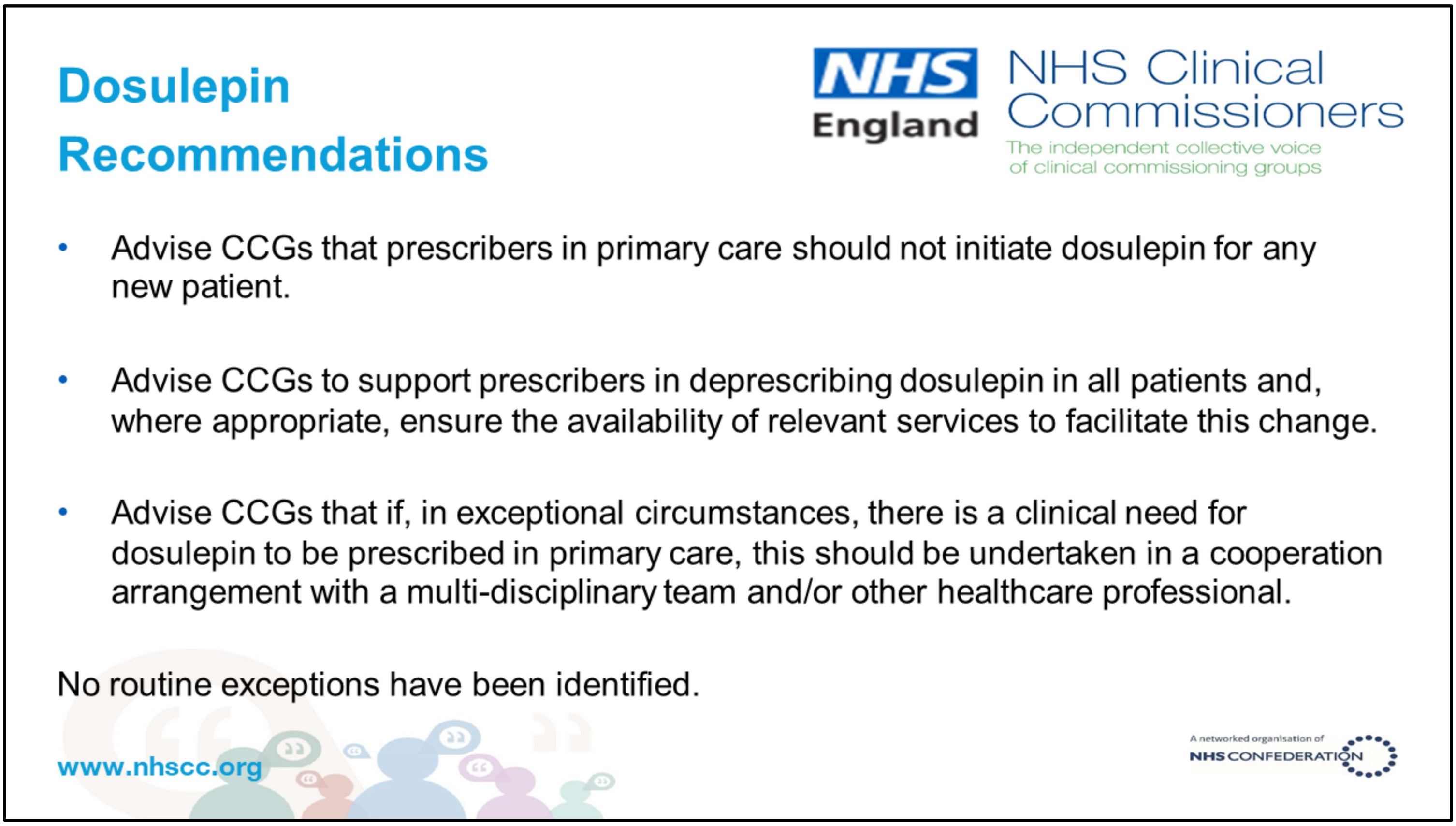

At this point display the dashboard data from OpenPrescribing for the CCG in question.
Explore the data
Discuss strategies to further reduce prescribing

Other Items

NHS England

NHS Clinical Commissioners
The independent collective voice of clinical commissioning groups

- Liothyronine (including Armour Thyroid and liothyronine combination products)
- Lutein and Antioxidants
- Once daily Tadalafil
- Vaccines administered exclusively for the purposes of travel
- Herbal treatments
- Homeopathy

www.nhscc.org

A networked organisation of NHS CONFEDERATION

Please note that these medicines may be used off-label for indications not covered in this presentation.

At this point demo the dashboard for the CCG focusing on their top 3.

Implementation

Patient Leaflets

NHS England

NHS Clinical Commissioners

The independent collective voice of clinical commissioning groups

Changes to medicines or treatments prescribed on the NHS

The NHS will be asking doctors to stop or greatly reduce the prescribing of certain medicines or treatments from December 2017. This is because they are:

- Not as safe as other medicines OR
- Not as good (effective) as other medicines OR
- More expensive than other medicines that do the same thing OR
- Shouldn't be available on the NHS in some circumstances.

This document will explain the changes, why they are happening and where you can get more information and support.

What medicines are included in this change?

NHS England guidance recommends that the following medicines or treatments should not be prescribed any more or should only be prescribed in special circumstances. They are:

Co-proxamol	Dosulepin	Doxazosin MR
Fentanyl IR	Glucosamine and Chondroitin	Herbal treatments
Homeopathy	Lidocaine plasters	Liothyronine
Lutein and antioxidants	Omega 3 fatty acids	Oxycodone and naloxone combination
Paracetamol and tramadol combination	Perindopril arginine	Rubefacients*
Tadalafil once daily	Trimipramine	

*Not including non-steroidal anti-inflammatory drugs

If you are not sure if you are using any of these medicines, you can ask your GP surgery or local pharmacist.

Why does the NHS want to reduce prescribing of these medicines?

The NHS has to make difficult choices about what it spends money on and

certainly more than they need to. What we're trying to do is to reduce the differences in these levels of prescribing.

These changes aim to improve prescribing for patients across the country and save the NHS valuable resources that can be spent on other areas of patient care.

What about patients that need to take a medicine on this list?

The guidance says that there are rare circumstances where individual patients will still be prescribed a medicine on this list. The circumstances vary for each drug and GPs will need to talk to individual patients.

How did NHS England decide which medicines to add to this list?

Some of the medicines were already on a National Institute of Health and Care Excellence (NICE) 'do not do' list. This means they should not be prescribed. For the other medicines the following areas were considered carefully:

- The legal status of the medicine
- The safety and effectiveness of the medicines
- Guidance from professional/national organisations
- The other treatments available
- The cost of the medicine.

Where can I find more information and support?

- You can speak to your local pharmacist, GP or the person who prescribed the medication to you.
- National and local charities can offer advice and support.
- The Patients Association can also offer support and advice: www.patients-association.org.uk/ or call 020 8423 8999
- Healthwatch: www.healthwatch.co.uk

Find out more about the medicines that are being stopped or reduced: https://www.england.nhs.uk/medicines/items-which-should-not-be-routinely-prescribed/

A networked organisation of NHS CONFEDERATION

CCGs would be expected to take this into account in formulating local policies, and for prescribers to reflect local policies in their prescribing practice.

PrescQIPP Drop-List and other resources

NHS Clinical Commissioners
The independent collective voice of clinical commissioning groups

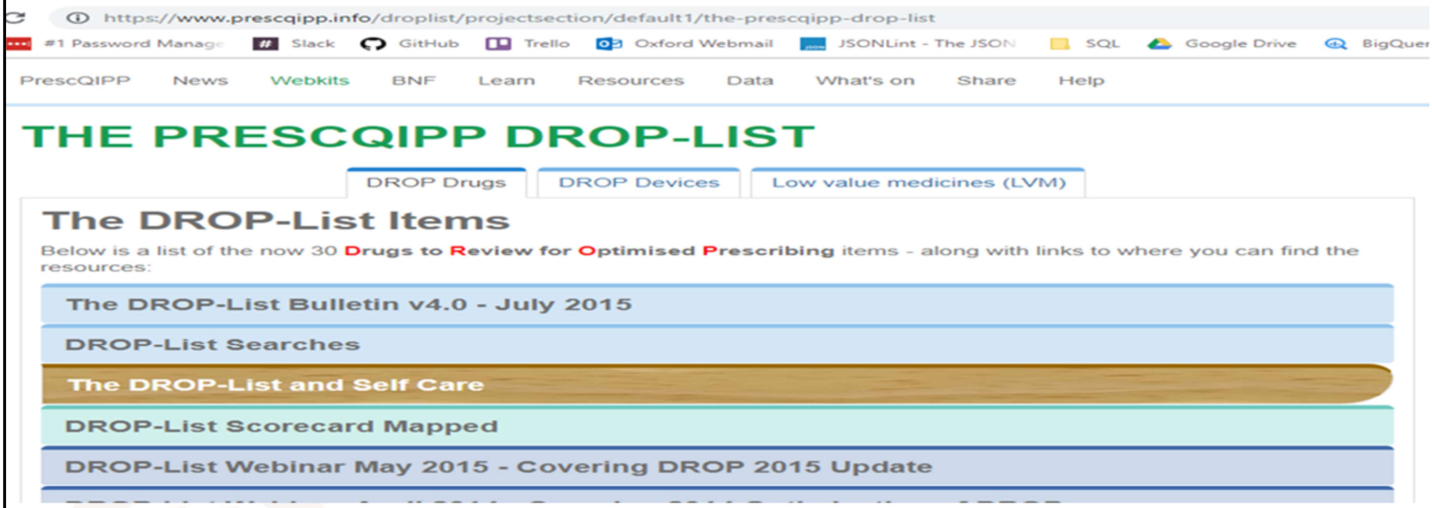
https://www.prescqipp.info/droplist/projectsection/default1/the-prescqipp-drop-list

#1 Password Manage Slack GitHub Trello Oxford Webmail JSONLint - The JSON SQL Google Drive BigQuer

PrescQIPP News Webkits BNF Learn Resources Data What's on Share Help

THE PRESCQIPP DROP-LIST

DROP Drugs | DROP Devices | Low value medicines (LVM)

The DROP-List Items

Below is a list of the now 30 **Drugs to Review for Optimised Prescribing** items - along with links to where you can find the resources:

- The DROP-List Bulletin v4.0 - July 2015
- DROP-List Searches
- The DROP-List and Self Care
- DROP-List Scorecard Mapped
- DROP-List Webinar May 2015 - Covering DROP 2015 Update

Reflections

NHS Clinical Commissioners
The independent collective voice of clinical commissioning groups

- Progress so far?
- Challenges?
- Support of stakeholders?
- Plan for next 12 months?

www.nhscc.org

A networked organisation of NHS CONFEDERATION

Approved/adopted the guidance?
Have you reviewed your formulary?
Have you reviewed all your guidance/schemes to see if they align?
Have you held an education event for GPs, pharmacists etc?
Have you discussed with your local secondary care services?
Have you programmed clinical systems? E.g. scriptswitch, OptimiseRx etc?
Have you reviewed all your guidance/schemes to see if they align?
Have patients been reviewed? If they havn't had reasonable reviews , it is unsafe according to WHO.

Low Priority Prescribing
Minor conditions

NHS England

NHS Clinical Commissioners
The independent collective voice of clinical commissioning groups

Conditions for which over the counter items should not routinely be prescribed in primary care (March 2018) – including recommendations on 35 minor conditions + vitamins & minerals

- Prioritise limited NHS funding
- Smarter use of resources
- Support the principle of self-care
- Reducing demand on general practice - It is estimated that there are 57 million GP consultations nationally a year for minor conditions at a total cost to the NHS of £2 billion. These appointments take up an average of one hour a day for every GP.
- Addressing increased price and other costs
- Reducing Variation

www.nhscc.org

A networked organisation of NHS CONFEDERATION

SPEND

Categories

NHS England

NHS Clinical Commissioners
The independent collective voice of clinical commissioning groups

35 conditions were categorised into one of the following categories:

- A condition that is self-limiting and does not require medical advice or treatment as it will clear up on its own; and/or

- A condition that is a minor illness and is suitable for self-care and treatment with items that can easily be purchased over the counter from a pharmacy.

And in the case of vitamins, minerals and probiotics, we classified these as:

Items of limited clinical effectiveness, where there is a lack of robust evidence for clinical effectiveness; however there may be certain indications where they may continue to be prescribed and these are outlined within the exceptions under the relevant item.

www.nhscc.org

A networked organisation of NHS CONFEDERATION

**Self-limiting conditions:**	**Items of limited clinical effectiveness**
1. Acute sore throat<br>2. Infrequent cold sore of the lip<br>3. Conjunctivitis<br>4. Coughs and colds and nasal congestion<br>5. Cradle Cap (Seborrhoeic dermatitis – infants)<br>6. Haemorrhoids<br>7. Infant Colic<br>8. Mild Cystitis	Probiotics<br>Vitamins and Minerals
**Minor conditions suitable for self care:**<br>9. Mild irritant dermatitis<br>10. Dandruff<br>11. Diarrhoea (Adults)<br>12. Dry Eyes/Sore tired eyes<br>13. Earwax<br>14. Excessive sweating (Hyperhidrosis)<br>15. Head lice<br>16. Indigestion and Heartburn<br>17 Infrequent constipation<br>18. Infrequent migraine<br>19. Insect bites and stings<br>20. Mild Acne<br>21. Mild dry skin<br>22. Sunburn due to excessive sun exposure<br>23. Sun protection	24. Mild to Moderate Hay fever/Seasonal Rhinitis<br>25. Minor burns and scalds<br>26. Minor conditions associated with pain, discomfort and/fever. (e.g. aches<br>and sprains, headache, period pain, back pain)<br>27. Mouth ulcers<br>28. Nappy Rash<br>29. Oral thrush<br>30. Prevention of dental caries<br>31. Ringworm/Athletes foot<br>32. Teething/Mild toothache<br>33. Threadworms<br>34. Travel Sickness<br>35. Warts and Verrucae

CCGs would be expected to take this into account in formulating local polices, and for prescribers to reflect local policies in their prescribing practice.

Exceptions

NHS England

NHS Clinical Commissioners

The independent collective voice of clinical commissioning groups

- Patients prescribed an OTC treatment for a long term condition (e.g. regular pain relief for chronic arthritis or treatments for inflammatory bowel disease)
- For the treatment of more complex forms of minor illnesses (e.g. severe migraines that are unresponsive to over the counter medicines)
- For those patients that have symptoms that suggest the condition is not minor (i.e. those with red flag symptoms for example indigestion with very bad pain)
- Treatment for complex patients (e.g. immunosuppressed patients)

Exceptions

NHS England
NHS Clinical Commissioners
The independent collective voice of clinical commissioning groups

- Patients on prescription only treatments.
- Patients prescribed OTC products to treat an adverse effect or symptom of a more complex illness and/or prescription only medications should continue to have these products prescribed on the NHS.
- Circumstances where the product licence doesn't allow the product to be sold over the counter to certain groups of patients. This may vary by medicine, but could include babies, children and/or women who are pregnant or breastfeeding. Community Pharmacists will be aware of what these are and can advise accordingly
- Patients with a minor condition suitable for self-care that has not responded sufficiently to treatment with an OTC product

Exceptions

NHS England

NHS Clinical Commissioners

The independent collective voice of clinical commissioning groups

- Patients where the clinician considers that the presenting symptom is due to a condition that would not be considered a minor condition.

- Circumstances where the prescriber believes that in their clinical judgement, exceptional circumstances exist that warrant deviation from the recommendation to self-care.

- Individual patients where the clinician considers that their ability to self manage is compromised as a consequence of medical, mental health or significant social vulnerability to the extent that their health and/or wellbeing could be adversely affected, if reliant on self-care. To note that being exempt from paying a prescription charge does not automatically warrant an exception to the guidance. Consideration should also be given to safeguarding issues.

Implementation

Patient Leaflets

NHS England

NHS Clinical Commissioners

The independent collective voice of clinical commissioning groups

A toolkit of resources to facilitate CCGs to implement the recommendations and support discussions between patients and their healthcare professionals including:

- Patient information leaflet (including an Easy Read version)
- Patient information sheet

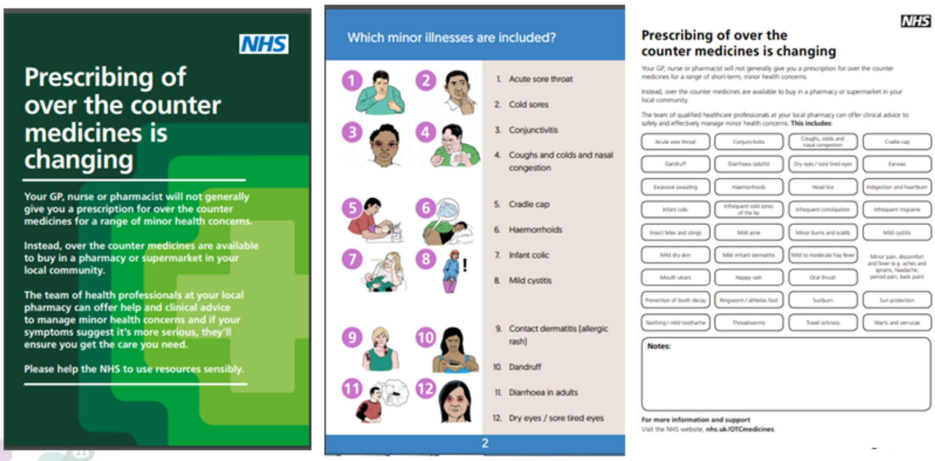

www.nhscc.org

CCGs would be expected to take this into account in formulating local polices, and for prescribers to reflect local policies in their prescribing practice.

Implementation

Other resources & interventions

NHS England

NHS Clinical Commissioners

The independent collective voice of clinical commissioning groups

- Quick reference guide
- Webinars & events e.g. care homes webinar 18 Sept
- Updates to NHS Choices – nhs.uk/OTCmedicines

Available via PrescQIPP

- Summer infographics poster highlighting the spend on sunburn and sun creams, insect bites, travel sickness mild hay fever
- Data resources

www.nhscc.org

A networked organisation of NHS CONFEDERATION

Implementation

In development…

NHS England

NHS Clinical Commissioners

The independent collective voice of clinical commissioning groups

- OTC dashboard
- Review of e-learning opportunities for prescribers
- Guide for GPs on the legal status of medicines to treat conditions in guidance
- Guide and implementation tools for commissioners on self care in schools and nurseries

www.nhscc.org

A networked organisation of NHS CONFEDERATION

References & Resources

NHS England

NHS Clinical Commissioners

The independent collective voice of clinical commissioning groups

NHS England Implementation resources:

- 18 items
- Minor conditions

PrescQIPP resources

NHS Digital Prescribing Cost Analysis (2017)

www.nhscc.org

A networked organisation of NHS CONFEDERATION

NHS England

NHS Clinical Commissioners

The independent collective voice of clinical commissioning groups

- What else could NHS England and NHS Clinical Commissioners do to support CCG around medicines policy?

www.nhscc.org

A networked organisation of NHS CONFEDERATION

NHS England

NHS Clinical Commissioners

The independent collective voice of clinical commissioning groups

Questions not answered today can be sent to
england.medicines@nhs.net

Thank you

www.nhscc.org

A networked organisation of NHS CONFEDERATION
